# Dietary gangliosides rescue GM3 synthase deficiency outcomes in mice accompanied by neurogenesis in the hippocampus

**DOI:** 10.3389/fnins.2024.1387221

**Published:** 2024-07-25

**Authors:** Jin-ichi Inokuchi, Shinji Go, Akemi Suzuki, Osamu Nakagawasai, Takayo Odaira-Satoh, Lucas Veillon, Takahiro Nitta, Paul McJarrow, Hirotaka Kanoh, Kei-ichiro Inamori, Koichi Tan-No, Michael Collett

**Affiliations:** ^1^Division of Glycopathology, Institute of Molecular Biomembranes and Glycobiology, Tohoku Medical and Pharmaceutical University, Sendai, Japan; ^2^Forefront Research Centre, Graduate School of Science, Osaka University, Toyonaka, Japan; ^3^Division of Pharmacology, Faculty of Pharmaceutical Sciences, Tohoku Medical and Pharmaceutical University, Sendai, Japan; ^4^Fonterra Research and Development Centre, Palmerston North, New Zealand

**Keywords:** GM3 synthase deficiency, milk gangliosides, GM3, GD3, oral supplementation, neurogenesis, hippocampus, cognitive function

## Abstract

Ganglioside GM3 synthase is a key enzyme involved in the biosynthesis of gangliosides. GM3 synthase deficiency (GM3SD) causes an absence of GM3 and all downstream biosynthetic derivatives, including all the a-, b-, c-series gangliosides, commonly found in neural tissues. The affected individuals manifest with severe irritability, intractable seizures, hearing loss, blindness, and profound intellectual disability. It has been reported that oral ganglioside supplementation has achieved some significant improvements in clinical symptoms, growth parameters, and developmental and cognitive scores in GM3SD patients. To gain insight into the molecular mechanisms of this supplementation, we performed supplementation of oral bovine milk gangliosides to GM3 synthase-deficient mice from early weaning periods. The oral milk ganglioside preparations were dominated by GM3 and GD3 gangliosides. Oral milk ganglioside supplementation improved the decreased cognitive function observed in GM3 synthase-deficient mice. The improvement in cognitive function was accompanied by increased ganglioside levels and neurogenesis in the hippocampus in the supplemented animals.

## 1 Introduction

In 1998, the human GM3 synthase gene (*GM3S*) was cloned (Ishii et al., 1998); 6 years later, the GM3S deficiency (GM3SD) was first reported by A. H. Crosby’s group as an autosomal recessive infantile-onset epilepsy syndrome associated with developmental stagnation and blindness in Old Order Amish (Simpson et al., 2004). Biallelic pathogenic *ST3GAL5* variants led to the disrupted synthesis of a-, b- and c-series gangliosides and consequently to severe infantile-onset various neurological disorders (Table 1). Until today, GM3S deficiency has been found in many ethnic groups not only Old Order Amish (Simpson et al., 2004) but also French (Fragaki et al., 2013), Pakistani (Gordon-Lipkin et al., 2018), African American (Boccuto et al., 2014), Korean (Lee et al., 2016), Italian (Indellicato et al., 2019), Reunion Islander and Algerian (Heide et al., 2022), Saudi Arabian (Abdulkareem et al., 2023), and Chinese (Watanabe et al., 2023). Various *ST3GAL5* mutations and symptoms in patients with GM3S deficiency are summarized in Table 1. This disease can cause severe neurodevelopmental defects characterized by progressive microcephaly, intellectual disability, choreoathetosis, blindness, deafness, intractable seizures, and/or pigment changes. Common features of these patients with GM3 synthase deficiency are delayed motor development, delayed language development, and reduced intellectual and memory abilities.

**Table 1:** Clinical features of ST3GAL5 patients^a^

Patient descent	Genotype	Disorder											Reference
		Mc	Pd/r	MD	Ep	AEEG	SHI	VI	Ir	DD	Sc	AP	
Old Order Amish	c.862C > T R288* Homozygous	NR	38/38	8/8	38/38	8/8	NR	8/8	8/8	8/8	NR	27/38	Simpson et al. (2004) and Wang et al. (2013)
Old Order Amish	c.862C > T R288* Homozygous	50/50	50/50	42/50	36/50	31/32	15/15	10/13	35/50	50/50	14/50	Few	Bowser et al. (2019)
French	c.862C > T R288* Homozygous	NR	2/2	2/2	2/2	NR	2/2	2/2	2/2	2/2	NR	NR	Fragaki et al. (2013)
Pakistani	c.862C > T R288* Homozygous	3/3	3/3	3/3	0/3	3/3	3/3	3/3	3/3	3/3	NR	2/3	Gordon-Lipkin et al. (2018)
African-American	c.1063G > A E355K Homozygous	4/4	4/4	3/3	1/4	NR	NR	0/1	1/1	4/4	NR	3/4	Boccuto et al. (2014)
Korean	c.584G > C, c.601G > A C195S,G201R Comp het	1/2	2/2	2/2	0/2	NR	NR	0/2	2/2	2/2	NR	1/2	Lee et al. (2016)
Italian	c.1024G > A G342S Homozygous	2/2	1/1	1/1	1/1	1/1	1/1	1/1	1/1	2/2	NR	1/1	Indellicato et al. (2019)
Saudi Arabian	c.221 T > A V74E Homozygous	3/3	3/3	3/3	3/3	3/3	NR	3/3	3/3	3/3	NR	NR	Abdulkareem et al. (2023)
Chinese	c.1000delC, c.1214dupG R334Efs*15, V406Sfs*10 Comp het	NR	2/2	1/2	2/2	2/2	2/2	2/2	2/2	2/2	2/2	1/2	Watanabe et al. (2023)
Reunion Islander	c.740G > A G247D Homozygous; c.740G > A, c.1063G > A G247D, E355K Comp het	9/16	16/16	14/14	12/16	NR	8/15	5/12	10/13	16/16	NR	5/16	Heide et al. (2022)
Algerian	c.1255 T > C *419Rext*38 Homozygous												
Italian	c.1000C > T, c.1166A > G R334X, H389R Comp het; c.1166A > G H389R Homozygous												
Italian	c.1024G > A, c.1166A > G G342S, H389R Comp het												

^a^Mc, microcephaly; Pd/r, psychomotor delay/regression; MD, movement disorder; Ep, epilepsy; AEEG, abnormal EEG; SHI, sensorineural hearing impairment; VI, vision impairment; Ir, irritability; DD, developmental delay; Sc, scoliosis; AP, abnormal pigmentation; Comp Het, compound heterozygous; NR, not reported.

There have been several studies looking at the effects of exogenous and orally administered gangliosides on brain functions (Park et al., 2005; Liu et al., 2014; Reis et al., 2016; Wang et al., 2019). The supplementation of gangliosides to variously aged animals and humans and the outcomes measured (incorporation into plasma and brain; cognitive and disease/disorder states) were variable with some inconsistency. These results may be due to variations in the composition and amounts of gangliosides tested and the timing of feeding.

To solve these issues, the present study was designed to increase the amount of concentrated and purified complex milk lipids (CMLs) from milk given orally to normal weaned mice and GM3S KO mice. Two CMLs, SuperZ and GL500, were prepared with different amounts of gangliosides. As shown in Table 2, SuperZ contains a large amount of GD3 and GM3 is one-tenth of GD3. On the other hand, GL500 contains more GM3 than GD3. Pellets were prepared so that the daily oral intake of gangliosides by mice was approximately 18 mg/day (720 mg/kg bw/day) and fed from weaning at 3 weeks of age.

**Table 2:** Total analysis of ingredients of SuperZ and GL500.^a^

Component	g/100 g sample	
	GL500	SuperZ
Moisture	2.8	3.0
Lactose	2.3	2.2
Ash (minerals)	12.3	11.2
Total lipids	82.6	83.6
Neutral lipids	28.41	27.74
Glycerophospholipids	47.59	47.06
PC (phosphatidylcholine)	6.32	5.73
PI (phosphatidylinositol)	9.11	8.98
PS (phosphatidylserine)	12.62	12.71
LPC (lysophosphatidylcholine)	0.17	0.00
PE (phosphatidylethanolamine)	13.50	14.84
SM* (total sphingomyelin)	5.32	4.46
LPS (lysophosphatidylserine)	0.15	0.18
LPE (lysophosphatidylethanolamine)	0.40	0.16
Gangliosides	6.6	8.8
GM3	4.0	0.8
GD3	2.6	8.0
TN powder	1.10	1.17
Total (moisture + lactose + ash + total lipids)	100.0	100.0

^a^Lactose calculated by difference. Neutral lipids are calculated as Total lipids – phospholipids – gangliosides. SM* is the sum of sphingomyelin + dihydrosphingomyelin. LPC, LPS, and LPE are minor lysophospholipids derived from partial hydrolysis of PC, PS, and PE. Phospholipids comprised all the total nitrogen in the protein test (N × 6.38); there was no significant level of protein in these samples.

The memory assessment methods include the fear-conditioning test, passive avoidance test, and mazes such as the water maze and the eight radial maze. Unlike the fear-conditioning test or maze, the novel object recognition test (NORT) can be tested under more natural conditions; that is, the test can assess memory without introducing artificial rewards or punishments, such as electric shocks or food restrictions, that might affect behavior (Kudara et al., 2023). Our study aimed to determine whether GL500 or SuperZ diet feeding affected memory; the NORT allowed us to examine the memory performance of animals under conditions without external rewards or punishments. The NORT was tested, and ganglioside composition of the serum, liver, and brain regions was then measured. In addition, the effects of SuperZ and GL500 on neurogenesis in the hippocampus were also examined.

## 2 Materials and methods

### 2.1 Preparation of diets containing milk gangliosides GM3 and GD3 with different ratios

We utilized two bovine milk-derived powders called GL500 and SuperZ (Fonterra Co-operative Group Ltd) containing concentrated gangliosides GM3 and GD3 at different ratios. As indicated in Table 2, GL500 was enriched in GM3, and SuperZ was enriched in GD3. The rodent diet AIN-93G (CLEA Japan, Inc.^1^) was used as a basis for the diets. The supplementation target was to make diet pellets containing 18 mg gangliosides per 4 g (amounts of daily uptake by adult mice). Milk powders tend to weaken the hardness of pellets, but sufficient hardness can be achieved when the pellets contain 20% sucrose. We thereafter used AIN-93G containing 20% sucrose as the base diet. Pelletization involves a drying process that requires heating at 70°C for 4 h. As shown by thin-layer chromatography (TLC; Supplementary Figure S1), there were no signs of degradation or changes of GM3 and GD3 levels in the pellets compared to the respective original milk powder. Analysis of GM3 molecular species by LC–MS/MS demonstrated that molecules containing N-acyl chains C22:0, C23:0, C16:0, C24:0, and C21:0 are the major GM3 species (shown in Supplementary Figure S2). These results were consistent with the report by Fong et al. (2011).

### 2.2 Animals

**Figure 1:**
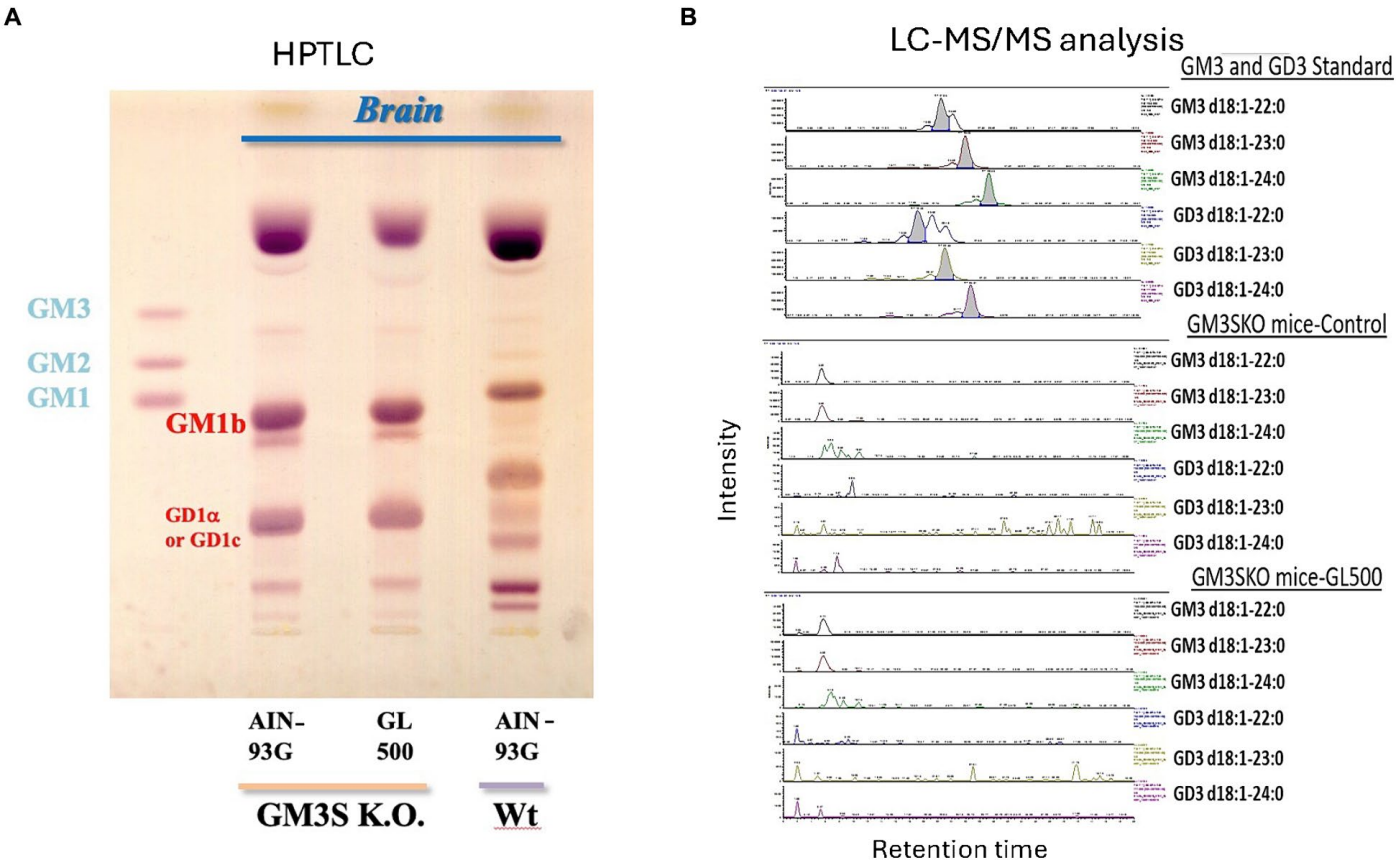
Brain gangliosides of wild-type (BALB/c) and GM3KO mice (♂) (*N* = 3/each group) fed a control diet (AIN-93G) or a diet supplemented with GL500 for 3 months from postnatal day 21. HPTLC analysis **(A)**. We do not have a standard for GD1α, but it has been reported that the brains of GM3S KO mice expressed GM1b and GD1α (Yoshikawa et al., 2009). There is a possibility to express GD1c, which migrates to the same position on HPTLC when we consider the metabolic pathway. LC–MS/MS analysis of GM3 and GD3 molecular species in the brain after feeding with GL500 **(B)**. There was no detectable milk-derived GM3 and GD3 in the whole brain of GM3S KO mice after 3 months of feeding of GL500.

GM3S KO mice were generated as described previously (Tsukamoto et al., 2005). In brief, the second exon of the 7-exon GM3 synthase gene was deleted and replaced with the neomycin resistance gene. Mutant mice were maintained on a BALB/c background by heterozygous mating to generate littermate controls. In this study, although the number of mice in each group was small, the study was performed among littermates because the experiments were conducted during the weaning period, which is affected by child-rearing. GM3S genotypes were determined using the Southern blot and PCR analyses of genomic DNA isolated from tail biopsies. Primer pairs used for genotyping included 5′-GGAATCCATC-CCTTTTCTCACAGAG-3′ and 5′-TGAACTCACTTGGCATT-GCTGG-3′ for detection of the wild-type allele (exon 2) and 5′-ACTGGGCACAACAGACAATCGG-3′ and 5′-TGGATACTTTCTCGGCAGGAGC-3′ for the knockout allele (neomycin resistance gene). The GM3S KO (homozygotes) mice were unable to synthesize GM3 ganglioside and GM3-derived complex gangliosides such as a-, b- and c-series gangliosides and instead expressed o-series gangliosides in the brain such as GD1α or GD1c (Figure 1). Mice were housed under conditions of constant temperature (23 ± 1°C) and humidity (55 ± 5%), on a 12-h/12-h light–dark cycle (light from 07.00 to 19.00 h; dark from 19.00 to 07.00 h). The mice had free access to water throughout the experimental period; 3-week-old mice were then fed control (AIN-93G), GL500, or SuperZ diet for 2–3 weeks or 3 months. All animal handling procedures were approved by the Guide for Care and Use of Laboratory Animals at Tohoku Medical and Pharmaceutical University.

### 2.3 Novel object recognition test

Mice were individually habituated to the test box for 10 min over 3 consecutive days after 3 weeks or 3 months of feeding, in the absence of objects. On the fourth day, two objects were symmetrically fixed to the floor of the box, and each mouse was allowed to explore the box for 5 min (training session). Following a 24-h delay, mice were placed back in the box for 5 min where one of the familiar objects was replaced by a novel object (retention session). The percentage of exploratory preference (%EP) was calculated as the exploration time of the novel object (N) divided by the total exploration time of both novel (N) and familiar (F) objects [%EP=N/(N + F)*100].

### 2.4 Hearing tests by startle response

Startle responses were measured using the SR-LAB Startle Response System (San Diego Instruments). A Plexiglass cylinder was positioned on a piezoelectric transducer that detected the vibrations caused by the movements of the animals. Each test session began by placing a subject in the Plexiglass cylinder for 10 min to acclimatize to a background noise of 65 dB SPL.

To assess acoustic startle responses, each subject was exposed to five consecutive four blocks of eight acoustic stimuli of increasing intensity (70, 75, 80, 85, 90, 100, 110, and 120 dB SPL), each in 20-ms broadband burst and separated by a 40-s interval. The startle response was measured and recorded every 1 ms for 100 ms starting at the onset of each startle stimulus. Each experiment lasted ~30 min.

To measure startle responses to air puff, a 10-ms burst of compressed air (0.2 mPa) was delivered to the dorsal side of each animal through a pipe located 2 cm above the subject. Bursts were repeated five times per animal, and responses were recorded as above.

For each experiment, statistical significance within each group was estimated using an F-test followed by Student’s *t*-test or the Aspin–Welch test.

Motor coordination and balance were measured using the Rota-Rod test. Mice were placed on a rotating rod (3 cm diameter; Neuroscience) with a non-skid surface, and the latency to fall was measured for up to 2 min. Rotation speed was 5, 10, and 15 rpm, and it increased 5 rpm every day. First, each mouse was trained until it achieved a criterion of staying on the rod for 2 min, three times in a row, at 5 rpm. The test was performed at 10 and 15 rpm, three times, for 2 days. The balance beam test was performed by using a 60-cm-long horizontal wooden dowel (1 cm diameter) placed at a height of 50 cm above a padded surface.

### 2.5 Analysis of GSLs

Lyophilized tissue was extracted twice with chloroform/methanol (2:1 and 1:1, v/v, consecutively). Total lipids were separated into acidic and neutral fractions on DEAE-Sephadex A-25 anion-exchange columns (GE Healthcare Life Sciences, Tokyo, Japan). Acidic and neutral lipids were de-esterified by mild alkaline hydrolysis and desalted using a Sep-Pak C18 Cartridge (Waters; Milford, MA). Acidic and neutral GSLs (protein equivalents of 1,000 μg and 200 μg, respectively) were spotted on HPTLC plates, developed with chloroform/methanol/0.2% CaCl_2_ (55:45:10, by vol) and chloroform/methanol/water (60:25:4, by vol), respectively, and then visualized by orcinol/sulfuric acid staining.

### 2.6 Mass spectrometric (MS) analysis

The LC–MS/MS analysis was performed using the method described previously (Veillon et al., 2015; Go et al., 2017). Neu5Ac GM3 (d18:1-[^13^C]16:0) was added to acidic GSL samples as the internal standard. GM3 molecular species were quantified using HPLC coupled with electrospray ionization tandem mass spectrometry (MS/MS) in multiple reaction-monitoring negative ionization mode. The triple-stage quadrupole (TSQ) Vantage AM instrument (Thermo Fisher, Waltham, MA) was calibrated by directly infusing a mixture of GM3 species extracted from milk, and all ion source parameters and ionization conditions were optimized to improve sensitivity. GSLs were dissolved in methanol, injected onto an HPLC pump (Accela 1,250, Thermo Fisher), and separated using a Develosil carbon 30 column (C30-UG-3–1 × 50 mm, Nomura Co., Aichi, Japan). The gradient program started with 100% solvent A (20% H_2_O, 50% 2-propanol, 30% methanol containing 0.1% acetic acid, and 0.1% ammonia) for 5 min and then ramped to 100% solvent B (2% H_2_O, 50% 2-propanol, 48% methanol containing 0.1% acetic acid, and 0.1% ammonia) over 30 min; 100% solvent B was maintained for 4 min, and then, the solvent was returned to 100% solvent A over 1 min and held there for 10 min. The flow was 50 μL/min throughout the chromatographic run, ~2,500 V potential was applied between the ion source and electrospray needle, and the carrier gas was nitrogen. Relative abundances of molecular species were assessed based on the relative percentage of internal standards. All GM3 molecular species may not necessarily have identical ionization efficiencies; however, because of the limited availability of pure molecular species standards, we assumed that all species have ionization efficiencies comparable with that of the internal standard. Thus, in evaluating relative abundances of molecular species, detected amounts are compared that may not necessarily represent absolute amounts (Veillon et al., 2015). The details for the assignment of ganglioside molecular species were described previously (Go et al., 2017).

### 2.7 Sialic acid analysis by fluorometric HPLC (DMB method)

Sialic acids were hydrolyzed from acidic GSL fractions using trifluoroacetic acid. An aliquot of the supernatant was lyophilized and then incubated with 1,2-diamino-4,5-methylene dioxybenzene (DMB) as described previously (Go et al., 2007). 1,2-Diamino-4,5-methylene dioxybenzene-labeled sialic acids were separated and detected using an HPLC system (JASCO; Tokyo, Japan) equipped with a reversed-phase C18 column (Wakopak Handy-ODS (4.6 mm × 250 mm); Wako Pure Chemical, Osaka, Japan).

### 2.8 Determination of neurogenesis in the dentate gyrus of the hippocampus

After 9 weeks of each diet feeding, 5-bromo-2′-deoxyuridine (BrdU) (50 mg/kg i.p.) was injected once daily for 5 days. After 3 months of feeding, animals were deeply anesthetized with sodium pentobarbital and intracardially perfused with phosphate-buffered saline (PBS), followed by paraformaldehyde (PFA). The brains were cut using a cryostat into 40 μm sections from bregma −2.20 mm to −2.80 mm, which included the dentate gyrus (DG). The sections were incubated with PBS containing 1% normal goat serum and 0.3% Triton X-100 (PBSGT) at room temperature. Then, the sections were incubated overnight with rat anti-BrdU monoclonal antibody (1:100) and mouse anti-NeuN monoclonal antibody (1:500). The sections were washed and then incubated with goat anti-rat IgG Alexa Fluor 568 (1:200) and goat anti-mouse IgG Alexa Fluor 488 (1:500) in PBSGT. Immunofluorescent images were analyzed using a confocal laser scanning microscope.

Three sections per mouse were used, and two images (left and right hemispheres, 640 × 640 μm) of the DG region of the hippocampus were obtained from each section. We calculated the BrdU/DCX- positive cells following methods outlined in our previous paper (Odaira et al., 2019). Positive cells were counted in the region observed under a fluorescence microscope. The positive cells in 2 images × 3 sections per mouse were added, and this total value is considered to be the total number of BrdU-/DCX-positive cells in the whole dorsal hippocampus. A total of six images were analyzed per mouse, and each group contained five to six mice.

### 2.9 Statistical analysis

The experimental results are presented as mean ± standard error of the mean (SEM). Significant differences were determined using one-way ANOVA, followed by a Tukey–Kramer test for multiple group comparisons. The criterion for a significant difference was set at *p* < 0.05.

## 3 Results

### 3.1 Effects of oral GL500 and SuperZ supplementation on ganglioside contents in the serum, liver, and brain regions in mice

Three-week-old BALB/c male mice were fed control pellets (Cont) or pellets containing GM3-rich GL500 or GD3-rich SuperZ for 2 weeks; 5-week-old mice were euthanized, and the serum, liver, and brain regions (the prefrontal cortex, hippocampus, thalamus, and hypothalamus) were collected. Samples in the same group were combined, and the ganglioside fractions were purified for HPTLC and LC–MS/MS analyses. The major ganglioside in the serum and liver of this mouse strain (BALB/c) is GM2. When the mice ate GL500 and SuperZ for 2 weeks from weaning, there was no detectable GM3 and GD3 in the serum (Supplementary Figure S3A) or liver using HPTLC (Supplementary Figure S4A) and LC–MS/MS (Supplementary Figure S4B) analysis. The major sialic acid species in BALB/c mice are NeuGc species, but the sialic acid species in cow milk are NeuAc species (Takamizawa et al., 1986; Puente and Hueso, 1993). Therefore, we measured the levels of NeuGc and NeuAc sialic acids in serum from mice fed control, GL500, or SuperZ diets. There was no increase in NeuAc species in GL500 or SuperZ groups (Supplementary Figure S3C). Thus, there was no signature of appearance of milk-derived gangliosides, or derivative components such as NeuAc, in mice receiving oral supplementation of GL500 or SuperZ.

**Figure 2:**
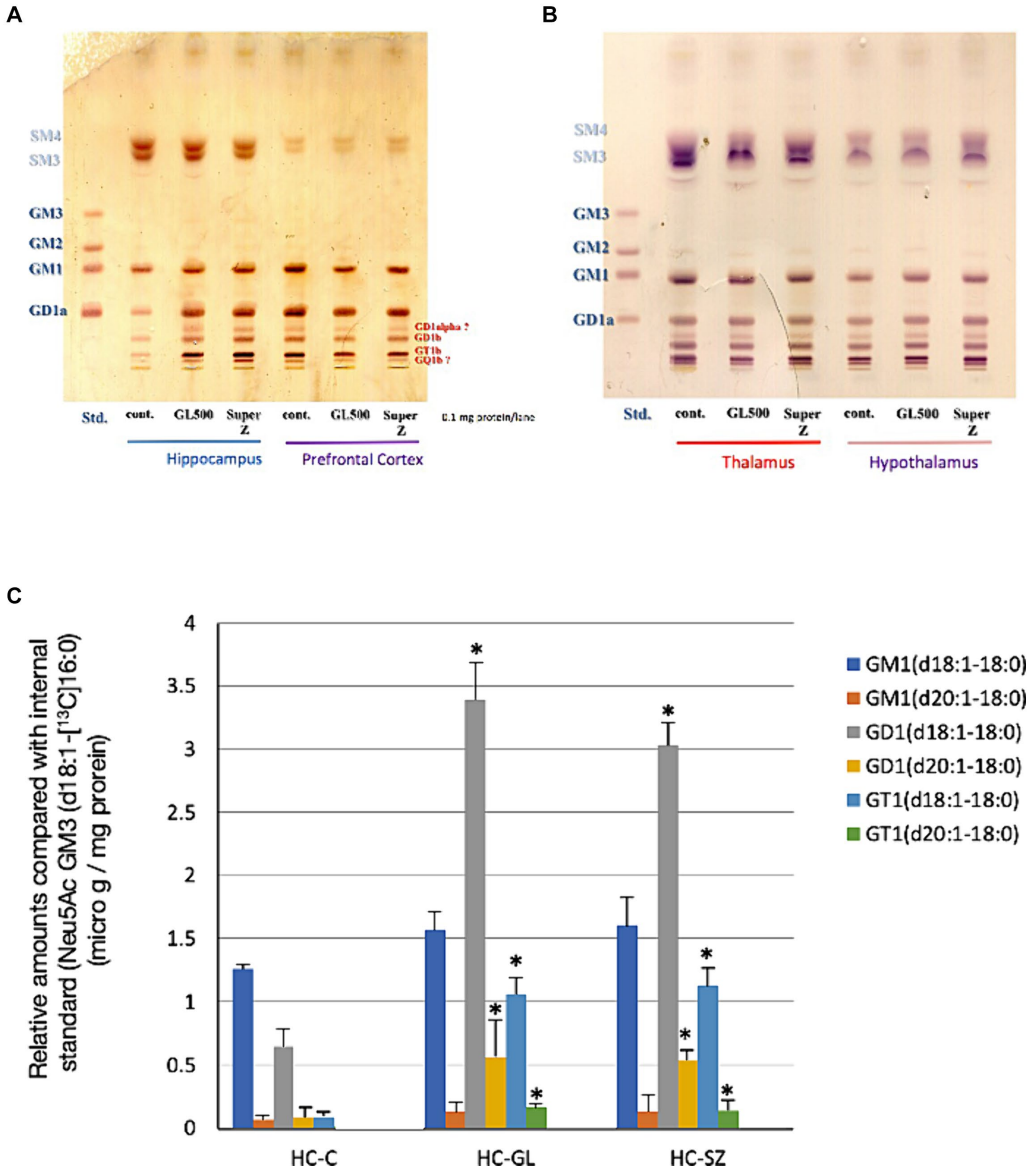
Selective increase of complex gangliosides in hippocampus from 3-week-old BALB/c mice (♂) (*N* = 3/group) fed for 2 weeks from postnatal day 21 with the control diet or a diet supplemented with GL500 or SuperZ. **(A,B)** Show HPTLC analyses of gangliosides in **(A)** the hippocampus and prefrontal cortex and **(B)** the thalamus and hypothalamus, while **(C)** shows the LC–MS/MS determination of complex ganglioside molecular species in the hippocampus (HC) of BALB/c mice (♂) fed with control diet (C), GL500 (GL), and SuperZ (SZ); an asterisk (*) indicates *p* < 0.05 vs. control (HC-C).

On the other hand, we observed a significant increase of complex gangliosides in the hippocampus of the same mice receiving GL500 or SuperZ but no increase in NeuAc or milk ganglioside components. As shown in Figure 2, the selective and significant increase in gangliosides in the hippocampus, especially GD1 and GT1 species, was detected in mice fed with GL500 or SuperZ (Figure 2A). Increase in GD1 (d18:1–18:0 and d20:1–18:0) and GT1 (d18:1–18:0 and d20:1–18:0) was confirmed by LC–MS (Figure 2C). As C23 GM3 was abundantly contained in both pellets (Supplementary Figure S2), the ceramide parts of milk GM3 and GD3 were not utilized directly for the increased synthesis of complex gangliosides in the hippocampus. There were no increases of such complex gangliosides in the other regions of the brain (the prefrontal cortex, thalamus, and hypothalamus) of the same mice (Figures 2A,B).

### 3.2 Influences of ganglioside diet feeding on NORT behavior by wild-type mice

**Figure 3:**
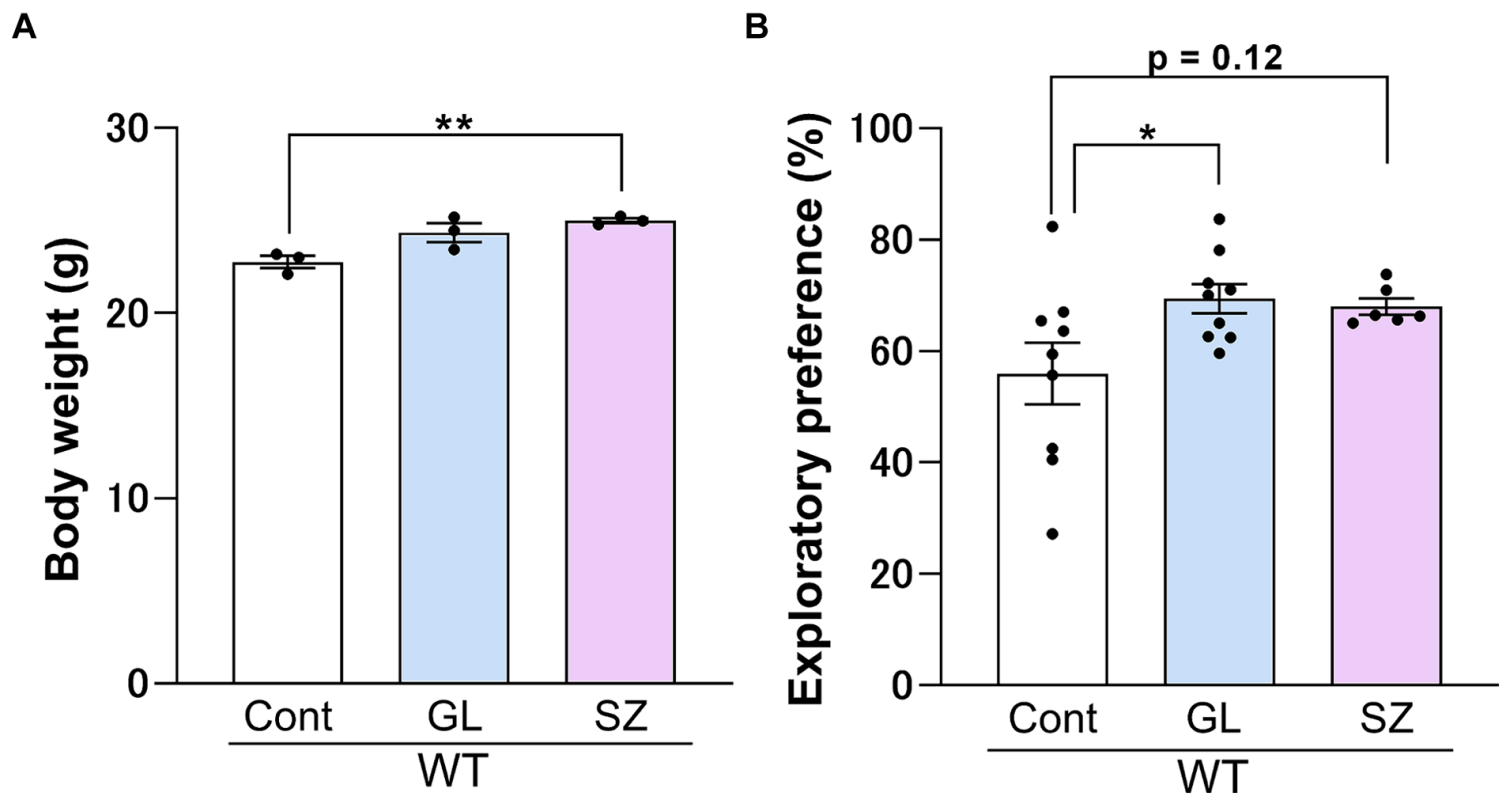
Influence of GL500 or SuperZ diet feeding on body growth **(A)** and cognitive function **(B)** of BALB/c mice (♂) fed from postnatal day 21 for 3 weeks with control diet (cont. diet) or diet supplemented with GL500 (GL) or SuperZ (SZ). **(A)** Shows an increase in the body weight of mice (*N* = 3 per group), and **(B)** shows the enhancement of the cognitive function of mice (*N* = 6–9 per group) fed with GL500 or SuperZ (asterisks indicate *p* < 0.05 vs. control diet-fed mice).

There were no significant differences in the exploratory preference for the training sessions after control, GL500, or SuperZ feeding for 3 weeks starting 3 weeks postpartum in WT mice (data not shown). Supplementation of GL500 to wild-type mice on early weaning periods significantly enhanced the exploratory preference in the retention session compared to the control diet (Figure 3B). SuperZ feeding also showed enhancements in growth (Figure 3A) and a trend in the exploratory preference in the retention session (*p* = 0.12) of WT mice (Figure 3B). These data indicated that supplementing the diet of WT mice with GL500 and SuperZ from the time of weaning (21 days after birth) for 2 weeks may enhance cognitive function by increasing gangliosides GD1 and GT1 in the hippocampus.

### 3.3 Influence of ganglioside diet feeding on GM3 synthase-deficient (KO) mice

**Figure 4:**
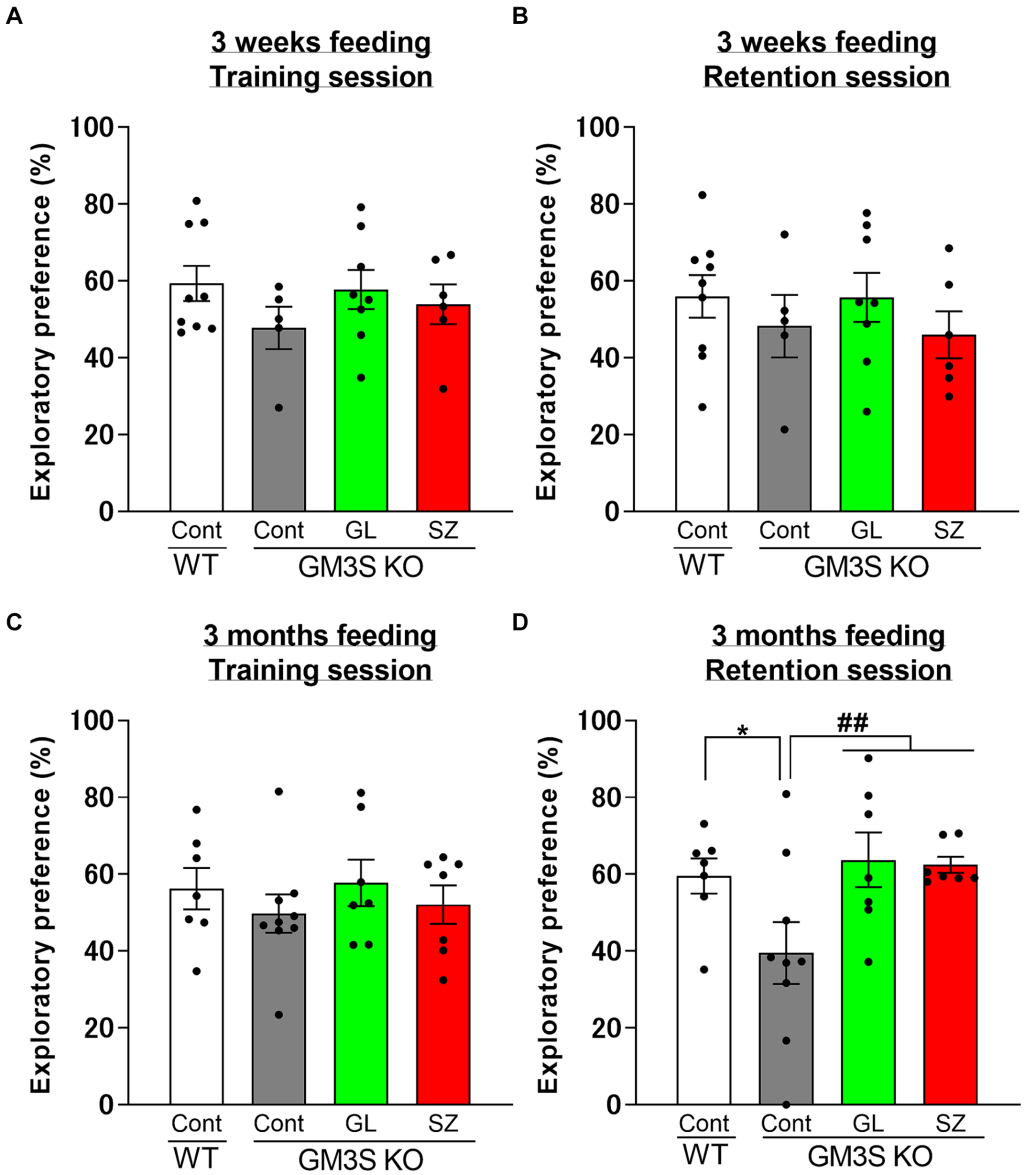
Influence of GL500 or SuperZ diet feeding for 3 weeks **(A,B)** and 3 months **(C,D)** from postnatal day 21 on cognitive function in WT and GM3S KO mice. Exploratory preferences for training **(A,C)** and retention **(B,D)** sessions are expressed as a percentage. *p < 0.05 vs. control diet fed WT mice. ^##^*p* < 0.01 vs. control diet fed GM3S KO mice. *n* = 5–9 per group.

In the training session, there were no significant differences in the exploratory preference between each group for 3 weeks or 3 months of feeding (Figures 4A,C). In the retention session, there were no significant differences in the exploratory preference between each group for 3 weeks of feeding (Figure 4B). Control diet-treated GM3S KO mice significantly decreased the exploratory preference on the retention session compared to control diet-treated WT mice (*p* < 0.05) after 3-month feeding (Figure 4D). GL500 (*p* < 0.01) and SuperZ (p < 0.01) treatments blocked the impairment of recognition memory in GM3S KO mice (Figure 4D).

However, analysis of gangliosides in whole brains of GM3S KO mice fed GL500 or SuperZ for 3 months did not confirm the presence of milk-derived GM3 and GD3 (Figure 1). Thus, ganglioside supplementation of GM3S-deficient mice did not normalize the ganglioside profile of the whole brain.

We reported for the first time that GM3S KO mice suffered from hearing loss (Yoshikawa et al., 2009); therefore, we performed “hearing tests” using the startle response on GM3S KO mice after 3 months of feeding GL500 and SuperZ. There was no improvement in hearing ability as assessed by startle response (Supplementary Figure S5).

### 3.4 Effects of GL500 and SuperZ on neurogenesis in the hippocampal dentate gyrus of GM3S KO mice

**Figure 5:**
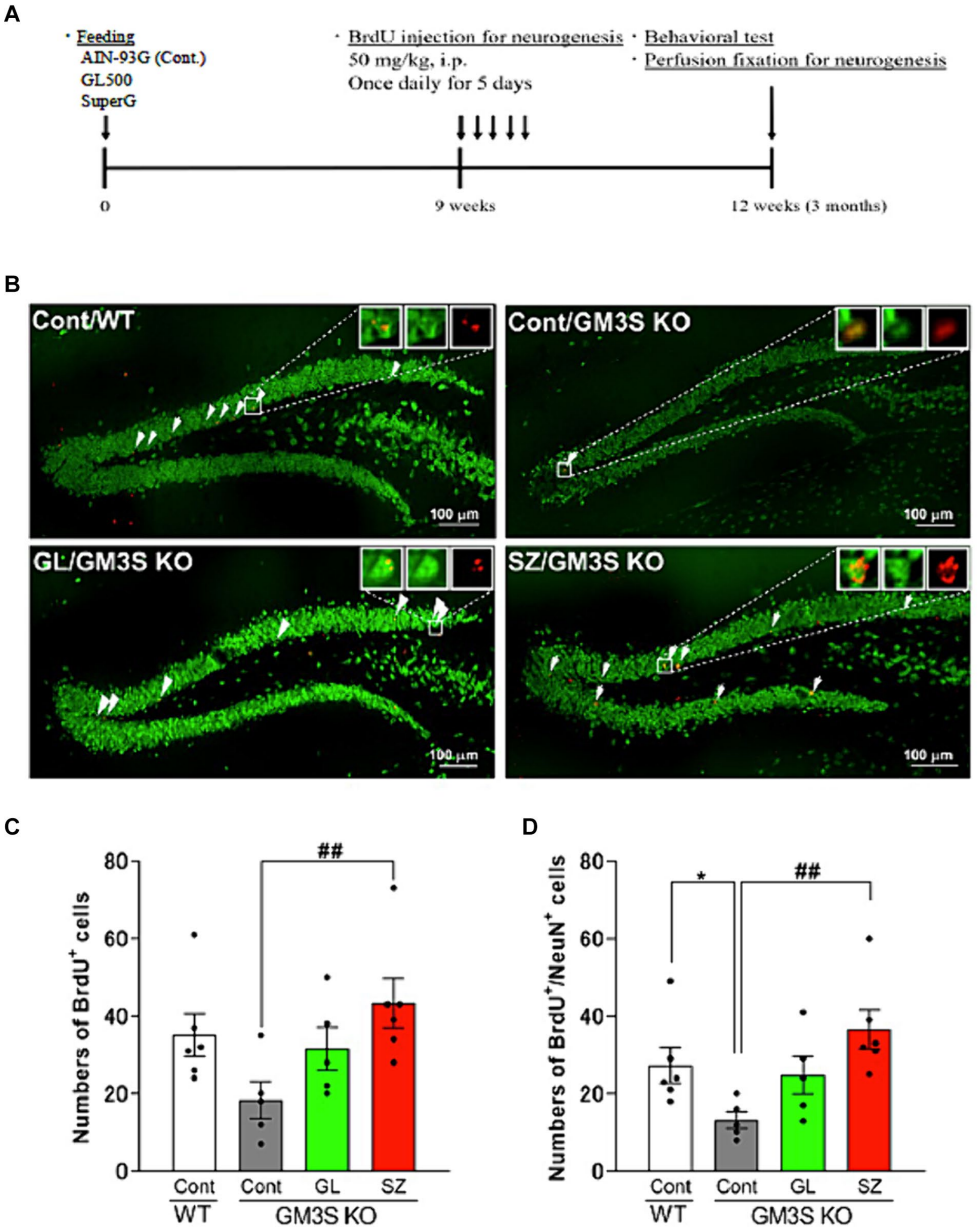
Effects of GL500 and SuperZ on neurogenesis in the hippocampal dentate gyrus of GM3S KO mice. **(A)** Shows the experimental protocol (details in Materials and methods). **(B)** shows immunohistochemistry images of brain slices stained with BrdU (red) and NeuN (green); the arrowhead point indicates double-positive cells. **(C,D)** Show quantification of the total number of BrdU **(C)** and BrdU-/NeuN-positive cells **(D)** in wild-type (WT) and GM3S KO mice fed 21 days postnatal for 3 months with control diet or control diet supplemented with GL500 (GL) or SuperZ (SZ); an asterisk (*) indicates *p* < 0.05 vs. control diet fed WT mice; a double hash (##) indicates *p* < 0.01 vs. control diet fed GM3S KO mice (*N* = 5–6 per group).

The experimental protocol is depicted in Figure 5A. BrdU and BrdU-/NeuN-positive cells in control diet-fed GM3S KO mice were fewer than in WT mice (BrdU-positive cells: *p* = 0.052, Figures 5B,C; BrdU-/NeuN-positive cells: *p* < 0.05, Figures 5B,D) at 3 months. In contrast, BrdU and BrdU-/NeuN-positive cells in the SuperZ-fed GM3S KO mice were significantly increased compared to control diet-fed GM3S KO mice (BrdU-positive cells: *p* < 0.01, Figure 5B,C; BrdU-/NeuN-positive cells: *p* < 0.01 Figures 5B,D). These results indicate that SuperZ feeding promotes neurogenesis in the hippocampal dentate gyrus of GM3S KO mice. GL500 feeding showed prevention of the decrease in neurogenesis in GM3S KO mice, but there was no increase.

## 4 Discussion

ST3GAL5 (GM3 synthase, GM3S) is located in the first step of the ganglioside biosynthetic pathway to the a-, b-, and c-series gangliosides commonly found in neural tissue. Inactivation of this enzyme by genetic mutation results in the loss of all complex gangliosides in the downstream a, b, and c pathways, causing severe damage to the central nervous system and other organs as shown in Table 1. B4GALNT1 (GM2 synthase, GM2S) synthesizes GM2 from GM3, but inactivation of the enzyme by mutation of the *B4GALNT1* gene results in only GM3 and GD3 gangliosides in the organism and none of the subsequent downstream a- and b-series gangliosides. GM2S deficiency was revealed when a mutation in the *B4GALNT1* gene was identified as the cause of hereditary spastic paraplegia 26 (SPG26) (Harlalka et al., 2013). Symptoms of SPG26 are substantially reduced compared to those seen in GM3S deficiency, suggesting that the severe symptoms of GM3S deficiency are mainly due to the additional loss of GM3 and GD3. GM3 and GD3 are known to be in higher concentrations and associated with early stages of neural development.

Therefore, we planned to investigate the effects of large oral doses of GM3 and GD3 on the central nervous system of weanling mice. The effects of oral administration of these gangliosides on learning performance have been studied in the past, but there are a number of variables, for example, dose, ganglioside composition, delivery method, animal age and developmental stage, and subsequently, the pharmacological effects on memory improvement also varied. In the present study, we performed NORT in wild-type and GM3 synthase-deficient mice orally ingesting 720 mg/kg bw/day of these gangliosides using two milk-derived formulations, GL500 and SuperZ, with different GM3 and GD3 ratios, prepared by Fonterra Cooperative Group Ltd. Our study utilizing NORT has revealed cognitive dysfunction in GM3S KO mice after 3 months of control diet feeding. This finding is consistent with a previous report that used the Y-maze in GM3S KO mice with C57/BL6 background (Niimi et al., 2011). These data suggest that GM3 may play a significant role in cognitive function.

There is a limited number of humans with GM3 synthase deficiency and the outcomes of the deficiency are more debilitating than that in the mouse model. The major impacts for human GM3 synthase deficiency within the study of Wang et al. (2019) were in decreasing irritability, improving interaction, and improving growth rates when supplementation in the range of 0.5–2 g of ingredient/kg, equivalent to 5.5–22 mg ganglioside/kg bw/day.

The use of the mouse model allows the use of cognitive testing and brain sample collection, which is very hard to undertake in the human cohort, and allows for the cohort to be of the same age and weight and the intervention to be times at a specific developmental time. The purpose of this study was to investigate the mouse model mechanism of changes with supplementation beginning at 21 days. The use of the mouse model also allowed for a larger dose of 720 mg gangliosides/kg bw/day.

Wang et al. (2019) investigated 13 individuals at different ages ranging from 0 to 115 months at the beginning of supplementation. This present study in GM3S KO mice confirmed the lack of impact of supplemented gangliosides on the serum ganglioside levels as in Wang et al. (2019).

Neurogenesis in the hippocampal dentate gyrus significantly contributes to central neuroplasticity mechanisms such as cognitive function (Massa et al., 2011). To determine the change in hippocampal neurogenesis in GM3S KO mice, the animals were injected with BrdU. Anti-NeuN antibody was then used to identify mature neurons in the dentate gyrus area. The incorporation of BrdU into a cell indicated that it was cycling at the time of BrdU injection. In the present study, GM3S KO mice with the BALB/c background showed decreases in hippocampal neurogenesis. However, it has been previously reported that young ST3GAL5 KO mice with the C57BL/6 background were not impaired with respect to adult hippocampal neurogenesis (Tang et al., 2020). Mice of the C57BL/6 strain are known to have approximately twice as much ability for new cell proliferation in the hippocampus and for neural cell migration and differentiation compared to BALB/C strain mice (Pozniak and Pleasure, 2006; Kim et al., 2009). These differences in results may be due to strain differences in the mice used in the experiments.

The GL500 and SuperZ feeding protected against cognitive dysfunction in GM3S KO mice. Similarly, it has been reported that neurogenesis is reduced in the hippocampus of Alzheimer’s disease patients (Lazarov and Marr, 2010). An increase in adult-born neurons is associated with the improvement of memory-related behavior in rodents (Oh et al., 2013).

Considering these reports, the present finding suggests that the enhancement of neurogenesis in the hippocampus by GL500 and SuperZ may be associated with the improvement of cognitive dysfunction in GM3S KO mice.

A recent study reported that IP administration of synthetic GM1 ganglioside to GM3S^−/−^ and GM3S^−/+^ mice was effective in preventing memory loss and the development of Parkinson’s disease-like symptoms (Chowdhury et al., 2023). The details of the mechanism of the effect of synthetic GM1 on GM3S^−/−^ and GM3S^−/+^ mice are not clear. We expect that the protective effect of milk gangliosides (GM3 and GD3) on reversing the decrease in hippocampal neurogenesis seen in GM3S^−/−^ mice may be related to the protective effect of synthetic GM1 on neuronal function.

## 5 Conclusion and perspectives

Oral supplementation of GM3 and GD3 to wild-type mice enhanced body weight gain and increased learning and memory abilities during early weaning periods, suggesting that oral ganglioside supplementation may be beneficial for infantile nutrition. Interestingly, the selective increase in complex gangliosides (GD1 and GT1) in the hippocampus (but no other regions of the brain) was observed in ganglioside-supplemented mice. For GM3S KO mice, GM3 and GD3 supplementation could block the decrease of cognitive function supporting the usefulness of the therapy of GM3SD patients performed by Wang et al. (2019). There was no evidence for the presence of milk-derived GM3 and GD3 molecules in the serum, liver, and brain after 3 months of feeding to wild-type mice and GM3S KO mice.

These results suggest that metabolites of GM3 and GD3 including sialic acids, ceramides, and fatty acids may positively affect infantile growth and brain development and improve symptoms of neurological disabilities for GM3SD patients. The lack of evidence in this study (and others) that the components of the oral gangliosides are incorporated into the brain gangliosides raises the question of what the mechanism is to achieve the functional outcome; this should be considered in future work.

## Data availability statement

The datasets presented in this study can be found in online repositories. The names of the repository/repositories and accession number(s) can be found in the article/Supplementary material.

## Ethics statement

All animal-handling procedures had been approved by the Guide for Care and Use of Laboratory Animals at Tohoku Medical and Pharmaceutical University. The study was conducted in accordance with the local legislation and institutional requirements.

## Author contributions

J-iI: Conceptualization, Data curation, Formal analysis, Funding acquisition, Investigation, Project administration, Supervision, Writing – original draft, Writing – review & editing. SG: Data curation, Formal analysis, Investigation, Methodology, Writing – original draft, Writing – review & editing. AS: Data curation, Investigation, Methodology, Writing – review & editing. ON: Investigation, Writing – original draft, Data curation, Formal analysis, Methodology, Writing – review & editing. TO-S: Investigation, Writing – original draft. LV: Data curation, Formal analysis, Investigation, Methodology, Writing – review & editing, Writing – original draft. TN: Data curation, Formal analysis, Investigation, Writing – review & editing, Methodology. PM: Conceptualization, Data curation, Formal analysis, Methodology, Writing – original draft, Writing – review & editing. HK: Data curation, Investigation, Writing – review & editing, Formal analysis. K-iI: Data curation, Investigation, Writing – review & editing. KT-N: Conceptualization, Data curation, Writing – review & editing, Investigation, Supervision. MC: Data curation, Formal analysis, Investigation, Writing – review & editing, Conceptualization, Project administration.

## Footnotes

^1^ https://www.clea-japan.com/products/special_diet/item_d0220
