# Supplementary material for: Dietary gangliosides rescue GM3 synthase deficiency outcomes in mice accompanied by neurogenesis in the hippocampus
Source: Front Neurosci. 2024 Jul 25;18:1387221. doi: 10.3389/fnins.2024.1387221 (PMC11308210; doi:10.3389/fnins.2024.1387221)
Supplement: Supplementary file 1 [file Data_Sheet_1.docx]

**Supplementary material**


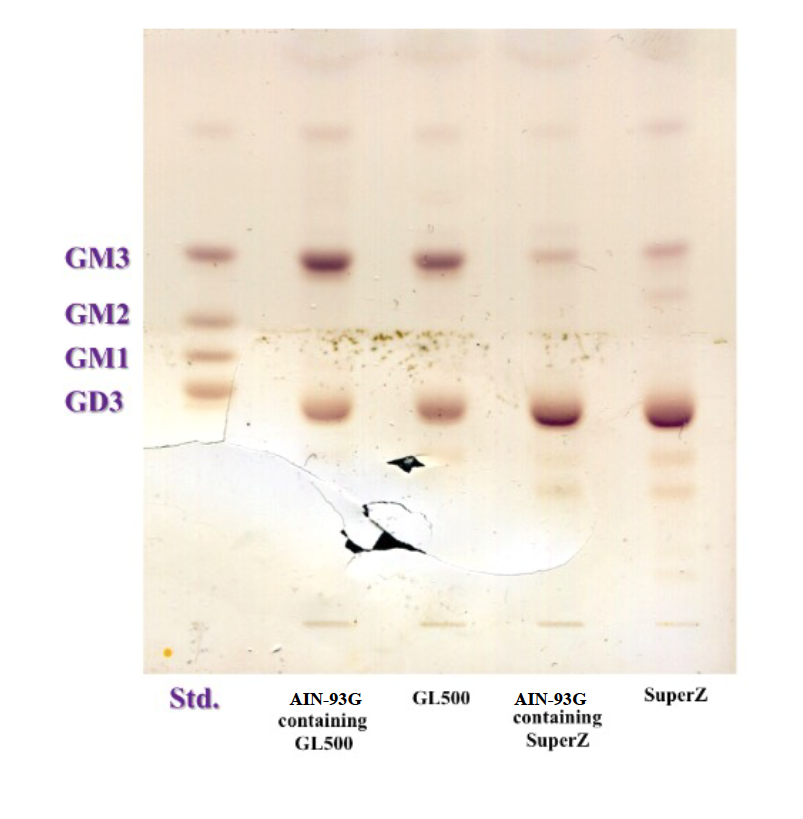


**Supplementary material Figure S1.** TLC analysis of glycosphingolipids in the pellets and the original powder. Stability of GM3 and GD3 after pelletization was examined by comparing with corresponding GM3 and GD3 levels in the original GL500 and SuperZ powders.

Starting materials for purification were: AIN-93G containing GL500:100 mg, GL500 original powder: 6.5 mg; AIN-93G containing SuperZ:100 mg, SuperZ original powder: 5.0 mg.


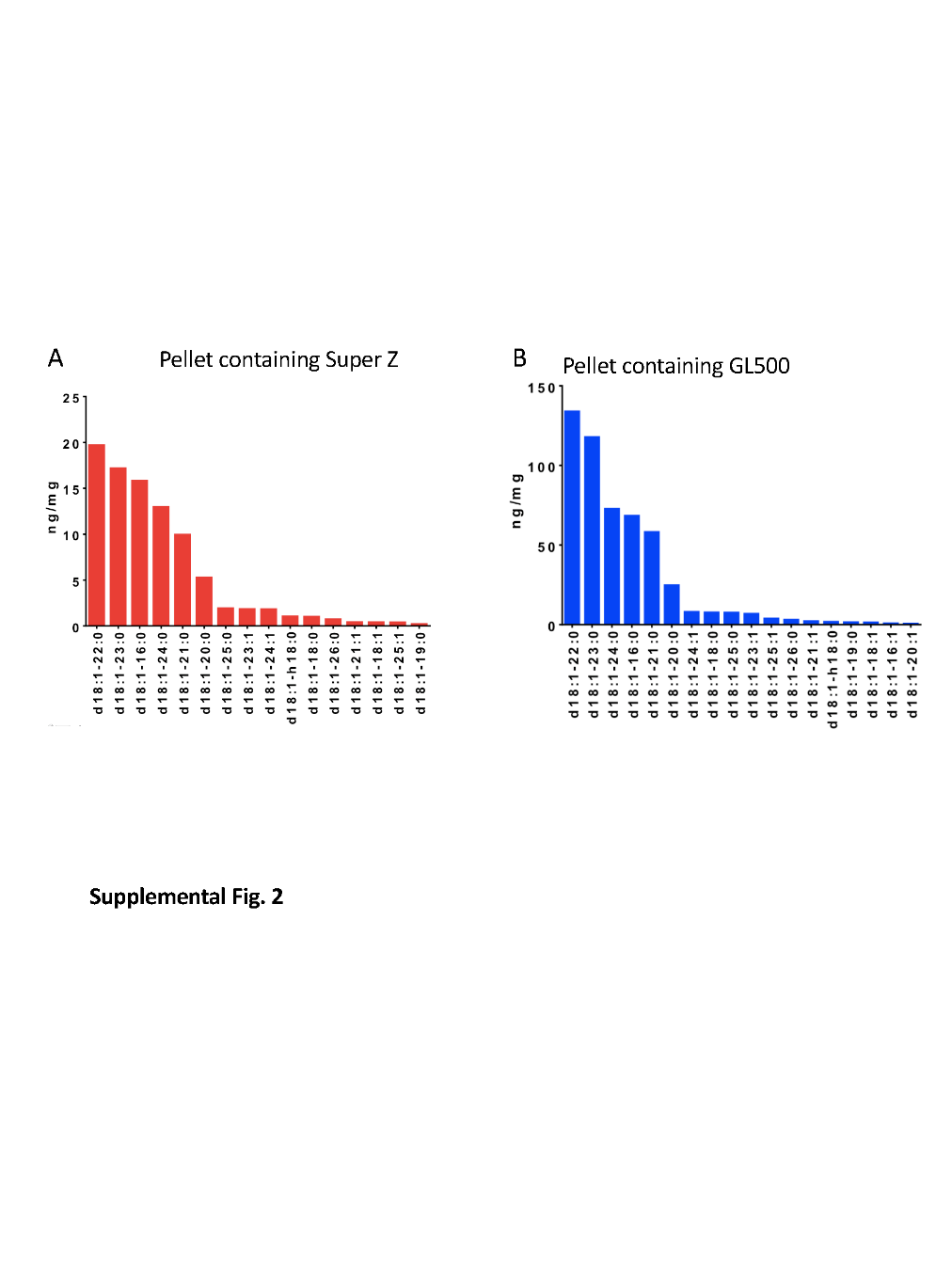


**Supplementary material Figure S2.** LC-MS/MS analysis of d18:1 GM3 molecular species in pellets containing SuperZ (A) or GL500 (B). GM3 molecular species in food pellets (ng/mg) were determined, using the internal standard NeuAcGM3(d18:1-[^13^C]16:0), as follows: GM3 molecular species (ng/mg)=(area of molecular species peak)/(area of internal standard peak)×(mass of internal standard added to food (ng))/(mass of food analyzed (mg)). Total d18:1 GM3 was calculated by taking the sum of all endogenous species measured. The evaluation comes with a caveat that not all d18:1 GM3 molecular species share identical ionization efficiencies; in this case, due to limited availability of pure GM3 molecular species standards, all species are assumed to have ionization efficiencies comparable with NeuAcGM3(d18:1-[^13^C]16:0).


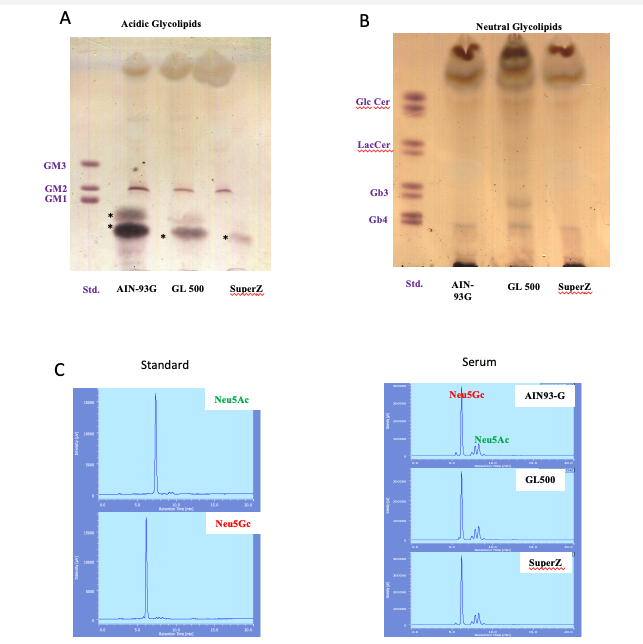


**Supplementary material Figure S3.** Serum glycosphingolipids of three weeks-old wild type BALB/c mice (♂) (N = 3/group) fed control diet or diet supplemented with GL500 or SuperZ for 3 months: HPTLC analysis of acidic lipid fraction (A) and neutral lipid fraction (B), GSLs purified from 200 µL serum was spotted in each lane (*non GSL contaminants during purification); HPLC analysis of Neu5GC and Neu5AC by DMB methods (C) was as described in the Materials and methods section in the main text. Serum from 3 mice of each group was combined for the analyses.

| A | B |
| --- | --- |
| 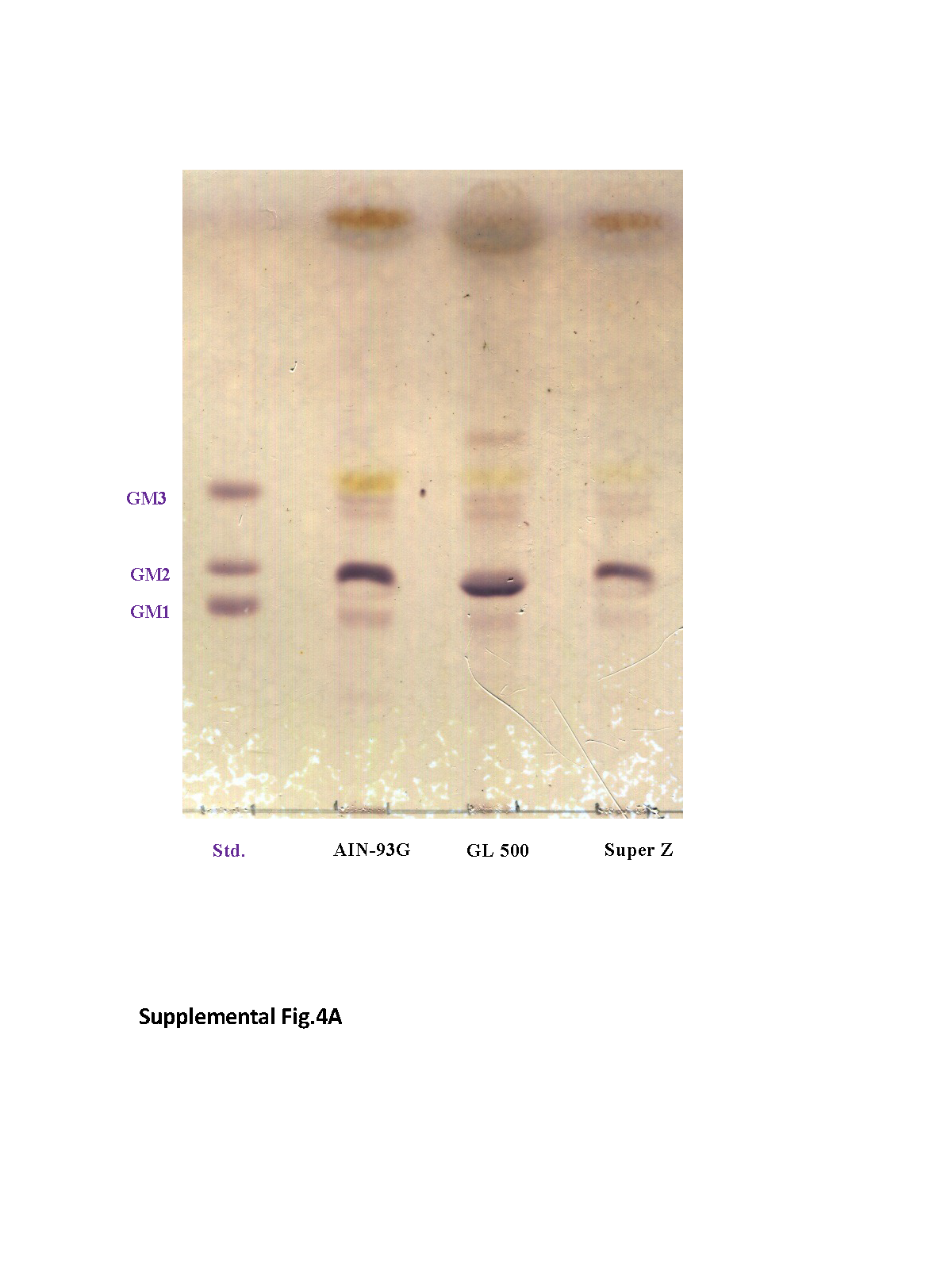 | 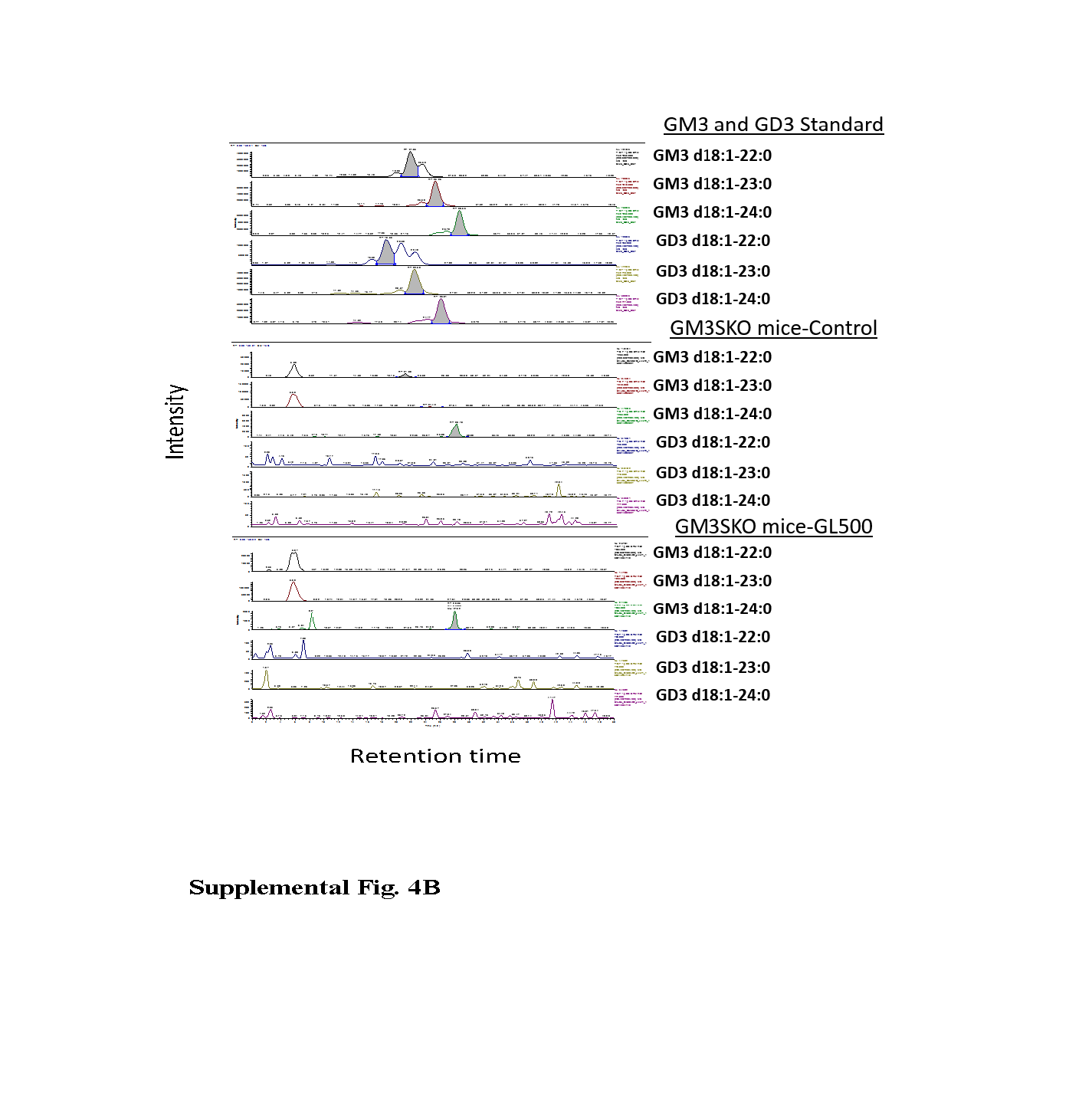 |
|  |  |

**Supplementary material Figure S4.** Liver gangliosides of three weeks-old wild type BALB/c mice (♂) (N = 3/group) fed control diet or diet supplemented with GL500 or SuperZ for 3 months (A) and LC-MS/MS analysis of GM3 and GD3 molecular species in liver after feeding with GL500 (B); liver from 3 mice of each group was combined and acidic lipid fractions purified from 10 mg protein were spotted in each lane for HPTLC analysis. LC-MS/MS analysis was as described in the Materials and methods section in the main text.


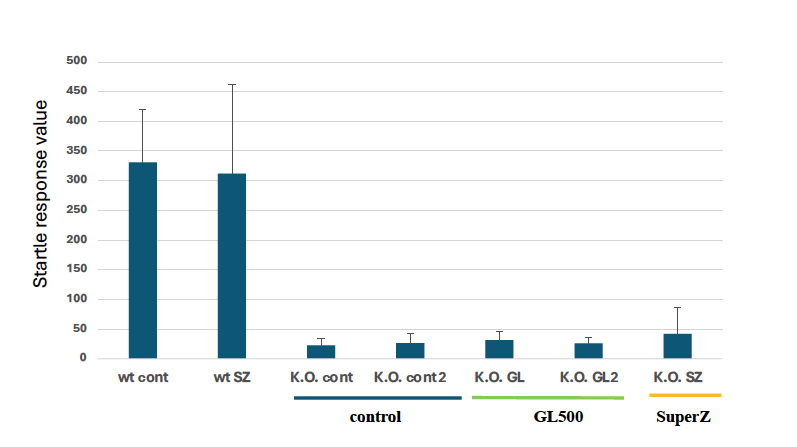


**Supplementary material Figure S5.** Hearing tests by startle response. The startle response was performed with control mice (wt cont and wt SZ) and GM3S KO mice after 3 months feeding control diet or diet supplemented with GL500 or SuperZ as described in the Materials and methods section in the main text. There was no improvement of hearing ability of GM3S KO mice after 3 months feeding GL500 or SuperZ.
